# A longitudinal study of *Blastocystis* in dairy calves from birth through 24 months demonstrates dynamic shifts in infection rates and subtype prevalence and diversity by age

**DOI:** 10.1186/s13071-023-05795-0

**Published:** 2023-06-02

**Authors:** Monica Santin, Aleksey Molokin, Jenny G. Maloney

**Affiliations:** Environmental Microbial and Food Safety Laboratory, Agricultural Research Service, United States Department of Agriculture, Beltsville, MD USA

**Keywords:** Blastocystis, Subtypes, Longitudinal study, Prevalence, Next-generation amplicon sequencing

## Abstract

*Blastocystis* is a common microeukaryotic intestinal parasite in humans and other animal hosts globally. However, no large-scale longitudinal study has ever been conducted for *Blastocystis*. To understand patterns of infection prevalence and subtype diversity and their relationship with host age, we have conducted the most comprehensive longitudinal study of *Blastocystis* infection ever performed. Dairy calves from a herd located in Maryland, USA, were followed from birth through 24 months of age, and 990 individual fecal samples from 30 calves were collected over the study period, representing three age groups (pre-weaned, post-weaned, and heifer). All samples were screened for *Blastocystis* via PCR, and subtype determination was performed using next-generation amplicon sequencing. Associations between age group and infection status were assessed using logistic regression analyses. *Blastocystis* infection prevalence increased with time, significant associations were observed between age groups and infection risk, and a cumulative prevalence of 100% was observed among the study population during the 24-month period. Thirteen previously reported subtypes (ST1-6, ST10, ST14, ST21, ST23–26) and one potentially novel subtype were observed. Diversity within ST10 supports the need for division of the subtype into new subtype designations. Associations between subtype and age group were explored, and relationships between subtypes and infection chronicity are described. While subtype diversity increased with age in the study population, distinct patterns of individual subtype prevalence and chronicity were observed, supporting the importance of subtype discrimination in studies of host infection and disease. The data from this study represent a significant advance in our understanding of *Blastocystis* infection dynamics within a single host population over time and can be used to inform future studies of *Blastocystis* epidemiology in both humans and other animal hosts.

**Graphical Abstract:**

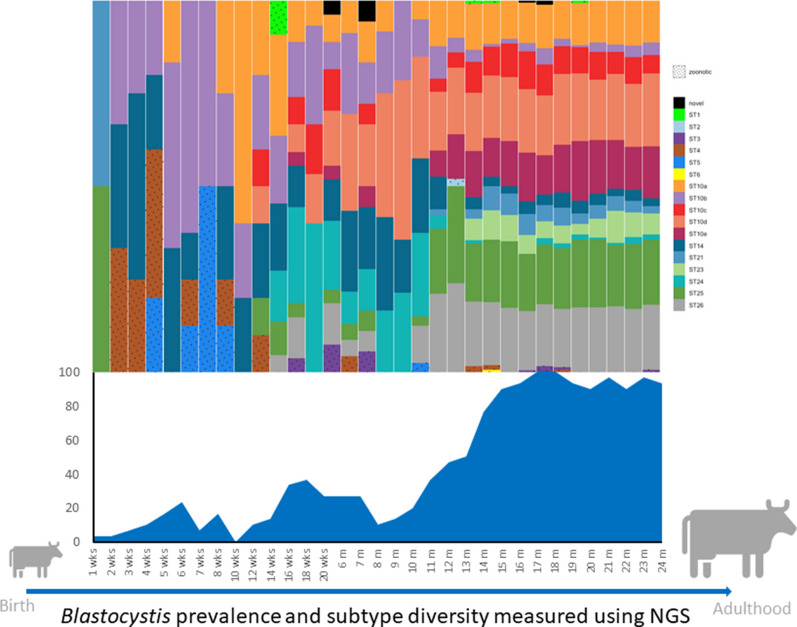

**Supplementary Information:**

The online version contains supplementary material available at 10.1186/s13071-023-05795-0.

## Background

*Blastocystis* is one of the most common intestinal parasites reported in humans and other mammalian and avian species globally [1, 2]. Despite being so common, the complex biology and epidemiology of *Blastocystis* have yet to be fully elucidated. While *Blastocystis* is thought to cause intestinal and extra-intestinal clinical manifestations in some patients, asymptomatic carriage is frequently observed, making the pathogenic potential and conditions under which *Blastocystis* may cause pathogenesis topics of interest and debate [3–6]. Additionally, the chronicity of infection remains unclear, as little data from longitudinal studies exist to assist in distinguishing chronic infection from reinfection from environmental exposure. Environmental exposure to the cyst form of the parasite occurs following consumption of food or water contaminated by feces of infected hosts, but sources of contamination are also not well characterized [7–10].

*Blastocystis* can infect both humans and other animals, yet the full extent of the zoonotic potential of different genetic variants of the parasite has not been determined. Much of the molecular epidemiology of *Blastocystis* is based upon genetic differences in the small subunit ribosomal RNA (*SSU* rRNA) gene. Characterization of this gene has led to the designation and validation of 33 *Blastocystis* subtypes (ST) [11–17]. Among these subtypes, host specificity is low, and most subtypes are reported in a variety of hosts [1]. However, some subtypes are commonly associated with certain animal groups. Subtype observations in humans provide an excellent example of the complicated nature of determining subtype host specificity. Subtypes 1–4 are the most common subtypes reported in humans, but an additional 11 STs (ST5, ST6, ST7, ST8, ST9, ST10, ST12, ST14, ST16, ST23, and ST35) have also been observed in samples from humans with varying frequencies [17–23]. All of these subtypes, except the recently described ST35, have also been reported in other animal hosts, indicating the potential for zoonotic transmission of *Blastocystis* [1, 24]. Aside from humans, other ST–host associations have also been noted. ST1, ST3, and ST5 are commonly reported in pigs [1]. ST6 and ST7 are commonly observed in samples from avian species, although it should be noted that these STs are frequently reported in mammalian hosts as well [22, 23, 25–27]. ST10 and ST14 are commonly observed in ruminant species [1]. Relationships between host and subtype are further complicated by the common occurrence of mixed-subtype infections within individual hosts [11, 28–31]. Molecular studies of *Blastocystis* subtype diversity from more hosts and more regions of the world which employ methods capable of revealing both prevalence and subtype diversity are needed to understand and define the full extent of subtype diversity and host specificity.

Longitudinal studies are an important tool in clarifying the complex epidemiology of *Blastocystis*, including understanding differences between chronic infection and reinfection as well as determining subtype occurrence patterns and trends over time. Yet, to date, no longitudinal studies employing molecular methods have followed a single population from birth to adulthood. Only four studies have used molecular methods to perform surveys of *Blastocystis* prevalence and ST distribution in humans over time. The first study sampled 10 individuals over different time periods ranging from 6 to 10 years and observed four individuals consistently positive for the same ST [32]. The second study followed 59 children from birth through 24 months, and testing for *Blastocystis* at week 1, week 8, month 12, and month 24 found only three study subjects positive for *Blastocystis* at 24 months [33]. The third study determined the presence and STs of *Blastocystis* in travelers before and after international travel and observed that most travelers’ carriage status was unchanged by travel [34]. The fourth study examined 679 stool samples from 125 toddlers attending day care centers in Spain to assess the presence of intestinal protist parasites, including *Blastocystis*, and found stable carriage of the same genetic variants, ST2, ST3, and ST4, in five children for up to 1 year [35]. Studies employing microscopy have also been performed to survey travel-associated infection and pre- and post-treatment infection clearance in humans [36–38]. Additionally, no longitudinal studies have been conducted in non-human hosts. Thus, there is a clear data gap in long-term studies of *Blastocystis* prevalence and ST diversity within the same host.

Cattle are an excellent system in which to explore *Blastocystis* epidemiology, as they are host to a wide variety of *Blastocystis* STs. Seventeen STs have been reported in cattle, while 15 STs have been reported in humans [1]. Cattle also exhibit age-associated differences in infection prevalence that are similar to those reported in humans, with infection rates increasing with age [39–42]. Thus, data from cattle could be used to inform potential trends in humans related to infection chronicity. Dairy cattle, which are often managed from birth in the same location, offer the additional advantage of following the same population of animals from birth through adulthood to better understand *Blastocystis* prevalence, subtype occurrence, and infection chronicity within a single population over time.

Longitudinal studies of *Blastocystis* are needed to assist in understanding the biology and epidemiology of this genetically diverse and cosmopolitan parasite. Such studies must employ molecular methods which can both define the prevalence of *Blastocystis* within the study population and delineate intra-host ST diversity. In the present study, fecal samples were collected at 33 time points from 30 purebred Holstein calves from birth through adulthood (24 months of age). All samples were tested for the presence of *Blastocystis* via polymerase chain reaction (PCR), and all positive samples were subtyped by next-generation amplicon sequencing (NGS) on an Illumina MiSeq system. This study is the most comprehensive and long-ranging longitudinal study ever conducted for *Blastocystis* and is the first longitudinal study of *Blastocystis* in cattle.

## Methods

### Sample collection and processing

This study was conducted under an animal use protocol approved by the Beltsville Area Animal Care and Use Committee. Fecal samples were collected from 30 purebred Holstein female calves from birth until 24 months of age at a dairy farm in Maryland, USA. Calves were housed individually in hutches from birth until 8 weeks of age. From 3 to 10 months of age, calves were housed in groups of 5–8 animals in pens which were partially covered by a roof. From 11 months until first calving at between 22 and 24 months, calves were on pasture. Feces were collected weekly from pre-weaned calves from 1 to 8 weeks of age (eight samples per calf), biweekly from post-weaned calves 10–20 weeks of age (six samples per calf), and monthly from heifers 6–24 months of age (19 samples per calf). A total of 33 samples per calf and 990 cumulative samples were collected. For sample collection, feces were collected directly from the rectum of each animal into a plastic cup. Cups were capped, labeled, and immediately placed in an insulated container packed with ice or cold packs. Parasite forms were concentrated from feces and DNA extracted as previously described [43].

### Molecular detection, NGS, library preparation, and bioinformatics analysis

Samples were screened, sequenced, and analyzed as described previously [28]. Briefly, all 990 samples were screened by PCR for *Blastocystis* using primers ILMN_Blast505_532F and ILMN_Blast998_1017R, which amplify a fragment of the *SSU* rRNA gene. These primers contain Illumina overhang adapter sequences on the 5′ end but are otherwise identical to Blast505_532F/Blast998_1017R [44]. Qubit fluorometric quantitation (Invitrogen, Carlsbad, CA, USA) was used to quantify final libraries prior to normalization, and a pooled library concentration of 8 pM with 20% PhiX Control was sequenced using Illumina MiSeq 600 cycle v3 chemistry (Illumina, San Diego, CA, USA). Paired-end reads were processed and analyzed with an in-house pipeline that uses the BBTools package v38.94 [45], VSEARCH v2.15.1 [46], and BLAST+ 2.11.0. Prior to operational taxonomic unit (OTU) assignment, read pairs were merged, filtered for quality and length, denoised, and checked for chimeric sequences. A 98% identity threshold was used for clustering and assignment of centroid sequences to OTUs, and OTUs with less than 100 sequences were discarded. OTUs were checked for chimeras a final time, and the remaining OTUs were blasted against *Blastocystis* references from the National Center for Biotechnology Information (NCBI). Raw FASTQ files were submitted to NCBI’s sequence read archive under project PRJNA927016 and accession numbers SRR23210482–SRR23210918. The nucleotide sequences generated using Illumina sequencing were deposited in GenBank under accession numbers OQ298847–OQ298915.

### Statistical analysis

Logistic regression analyses were used to test for significant associations between *Blastocystis* infection and age categories [pre-weaned (1–8 weeks), post-weaned (3–11 months), and heifer (12–24 months)]. Separate analyses were performed to compare differences between all three age categories and differences between animals < 12 months and ≥ 12 months. Statistical analyses were performed using R version 4.1.2 (R Core Team, 2021). *P*-values < 0.05 were considered statistically significant.

## Results

### Prevalence of *Blastocystis*

Among the 990 dairy heifer calf samples tested for the presence of *Blastocystis* in this study, there was an overall prevalence of 44.1% (437/990). The cumulative prevalence in the study population was 100%, with all 30 calves included in the study testing positive for *Blastocystis* at least once in the 24-month collection period. The number of *Blastocystis*-positive samples per calf ranged from 7 to 22, with an average of 14.6 positive samples per calf.

*Blastocystis* prevalence increased with age in the study population. Among the three age categories included in this study, pre-weaned calves (1–8 weeks) had the lowest overall prevalence at 10.8% (26/240) and lowest cumulative prevalence at 40% (12/30). Among post-weaned calves (3–11 months), overall prevalence was 21.1% (76/360) and cumulative prevalence was 83.3% (25/30). Among heifers (12–24 months), both overall and cumulative prevalence were highest at 85.9% (335/390) and 100% (30/30), respectively. Logistic regression analysis comparing *Blastocystis* infection between the pre-weaned and the post-weaned and heifer groups found significant associations with age (Table 1). Infection risk increased significantly with age, and the odds of infection were highest in the heifer group.

**Table 1:** Logistic regression analysis comparing *Blastocystis* infection status (any subtype or subtype combination, zoonotic subtypes, or single subtype/subgroup infections) between pre-weaned and post-weaned and heifer age groups

	Age category	Log odds	*P*-value	95% CI
*Blastocystis*	Post-weaned	0.79	0.001^c^	0.3, 1.3
	Heifer	3.91	9.06E−54^c^	3.4, 4.4
Zoonotic subtypes^a^	Post-weaned	−0.53	0.26	−1.4, 0.4
	Heifer	−0.02	0.97	−0.8, 0.8
ST10a^b^	Post-weaned	1.73	0.005^c^	0.5, 2.9
	Heifer	4.45	4.42E−14^c^	3.3, 5.6
ST10b^b^	Post-weaned	0.49	0.14	−0.2, 1.1
	Heifer	0.79	0.01^c^	0.2, 1.4
ST10c^b^	Post-weaned	15.90	0.98	−1334.6, 1376.5
	Heifer	18.92	0.98	−1341.6, 1379.5
ST10d^b^	Post-weaned	17.46	0.98	−1343.1, 1378
	Heifer	20.95	0.98	−1339.6, 1381.5
ST10e^b^	Post-weaned	14.79	0.98	−1345.8, 1375.3
	Heifer	19.89	0.98	−1340.7, 1380.4
ST14	Post-weaned	0.98	0.01^c^	0.2, 1.7
	Heifer	1.42	0.0001^c^	0.7, 2.1
ST24	Post-weaned	17.46	0.98	−1343.1, 1378
	Heifer	16.48	0.98	−1344.1, 1377
ST25	Post-weaned	1.69	0.11	−0.4, 3.8
	Heifer	6.83	1.34E−11^c^	4.9, 8.8
ST26	Post-weaned	16.28	0.98	−1344.3, 1376.8
	Heifer	20.95	0.98	−1339.6, 1381.5

CI confidence intervals

^a^Zoonotic subtypes included ST1–ST6

^b^ST10 was divided into five subgroups (ST10a, ST10b, ST10c, ST10d, and ST10e)

^c^Statistically significant

**Fig. 1:**
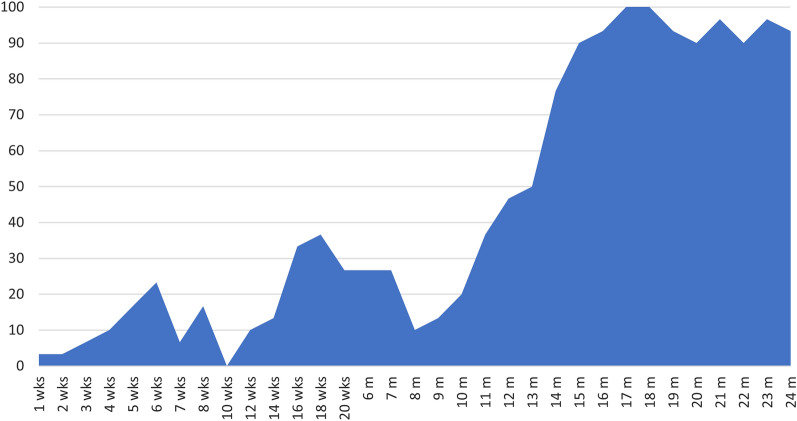
Point prevalence of *Blastocystis* in dairy heifer calves from 1 week to 24 months of age

However, *Blastocystis* prevalence did not increase consistently at each time point measured in the study (determined at weekly, biweekly, and monthly age intervals up to 24 months of age). Point prevalence data demonstrated periods of increase and decline in *Blastocystis* infection prevalence among dairy calves throughout the study period (Fig. 1). *Blastocystis* prevalence increased steadily from 3.3% at 1 week of age to 23.3% at 6 weeks of age. Following this peak in the pre-weaned age group, infection decreased to 0% by 3 months of age (10 weeks). Notably, 3 months was the only age group for which no positive samples were observed. Prevalence increased to 36.7% at 18 weeks and then remained at 26.7% until 7 months. At 8 months there was another sharp drop in prevalence to 10%. Following this final decrease, prevalence steadily increased and appeared to plateau between 15 and 18 months (Fig. 1). From 15 to 24 months, prevalence ranged from 90 to 100%, indicating a high prevalence and constant presence of *Blastocystis* in heifers in the study population.

### Molecular characterization of *Blastocystis* subtypes

Sixty-nine unique genetic variants of *Blastocystis* were found by NGS among the 437 *Blastocystis*-positive samples. Among those genetic variants, 13 previously reported STs (ST1–ST6, ST10, ST14, ST21, ST23–ST26) and one potentially novel ST were identified.

The potentially novel subtype observed in this study was represented by two unique genetic variants (Table 2). These two nucleotide sequences shared 99.6% sequence identity. Comparisons between the two nucleotide sequences representing the potentially novel ST and sequences available on GenBank were performed via BLAST. A single sequence reported as ST3 (note that the sequence shared only 96% sequence identity by BLAST with the closest match in GenBank, which was an ST3) (GenBank accession no. MW301904) shared between 98.7% and 99.1% sequence identity with the potentially novel ST sequences. The next best match on GenBank was also a sequence classified as ST3 (GenBank accession no. HQ909891), which shared between 96.7% and 96.3% sequence identity with the potentially novel ST sequences. The sequences observed in cattle representing the potentially novel subtype differ by ≥ 3% from any existing subtype; however, these comparisons are based on an approximately 480-base-pair (bp) region of the *SSU* rRNA gene. Unfortunately, DNA from samples that contained these sequences was not available to obtain full-length reference sequences of the *SSU* rRNA gene for the potentially novel ST. Without these data, designation of the potentially novel subtype as a new ST should be withheld. This sequence variant will be referred to as a potentially novel ST throughout this manuscript.

**Table 2:** Prevalence of *Blastocystis* subtypes and unique genetic variants observed among the 437 *Blastocystis*-positive calf specimens

Subtype^a^	No. of samples	No. of unique genetic variants	Percentage of positive samples	GenBank accession number
ST1	5	5	1.1	OQ298902; OQ298904; OQ298906; OQ298910; OQ298912
ST2	1	1	0.2	OQ298895
ST3	10	4	2.3	OQ298874; OQ298893; OQ298894; OQ298913
ST4	13	4	3.0	OQ298873; OQ298885; OQ298886; OQ298899
ST5	5	1	1.1	OQ298868
ST6	1	1	0.2	OQ298909
ST10a	230	5	52.6	OQ298850; OQ298857; OQ298896; OQ298897; OQ298905
ST10b	94	17	21.5	OQ298855; OQ298865; OQ298867; OQ298869; OQ298871; OQ298872; OQ298877; OQ298878; OQ298881; OQ298884; OQ298887;OQ298889; OQ298890; OQ298898; OQ298907; OQ298911; OQ298914
ST10c	143	3	32.7	OQ298859; OQ298861; OQ298883
ST10d	351	2	80.3	OQ298847; OQ298851
ST10e	229	4	52.4	OQ298854; OQ298870; OQ298908; OQ298915
ST14	97	8	22.2	OQ298856; OQ298876; OQ298879; OQ298882; OQ298888; OQ298892; OQ298900; OQ298901
ST21	73	3	16.7	OQ298858; OQ298880; OQ298891
ST23	98	1	22.4	OQ298852
ST24	56	2	12.8	OQ298860; OQ298864
ST25	319	3	73.0	OQ298849; OQ298853; OQ298866
ST26	325	3	74.4	OQ298848; OQ298862; OQ298863
Novel	5	2	1.1	OQ298875; OQ298903

^a^ST10 was divided into five subgroups (ST10a, ST10b, ST10c, ST10d, and ST10e)

### Blastocystis intra-subtype diversity

Intra-subtype diversity was observed for all STs found in multiple samples except for ST5 and ST23, with a single genetic variant identified in five and 98 positive samples for ST5 and ST23, respectively (Table 2). All other STs presented intra-subtype diversity. The five ST1-positive samples comprised five unique genetic variants, and both ST3 and ST4 had four unique genetic variants in the study population among 10 and 13 positive samples, respectively (Table 2). ST10 had the most intra-subtype diversity with 31 unique genetic variants. ST14 through ST26 ranged from two to eight unique genetic variants per subtype (Table 2). The potentially novel ST had two unique genetic variants among the five positive samples in the study.

**Fig. 2:**
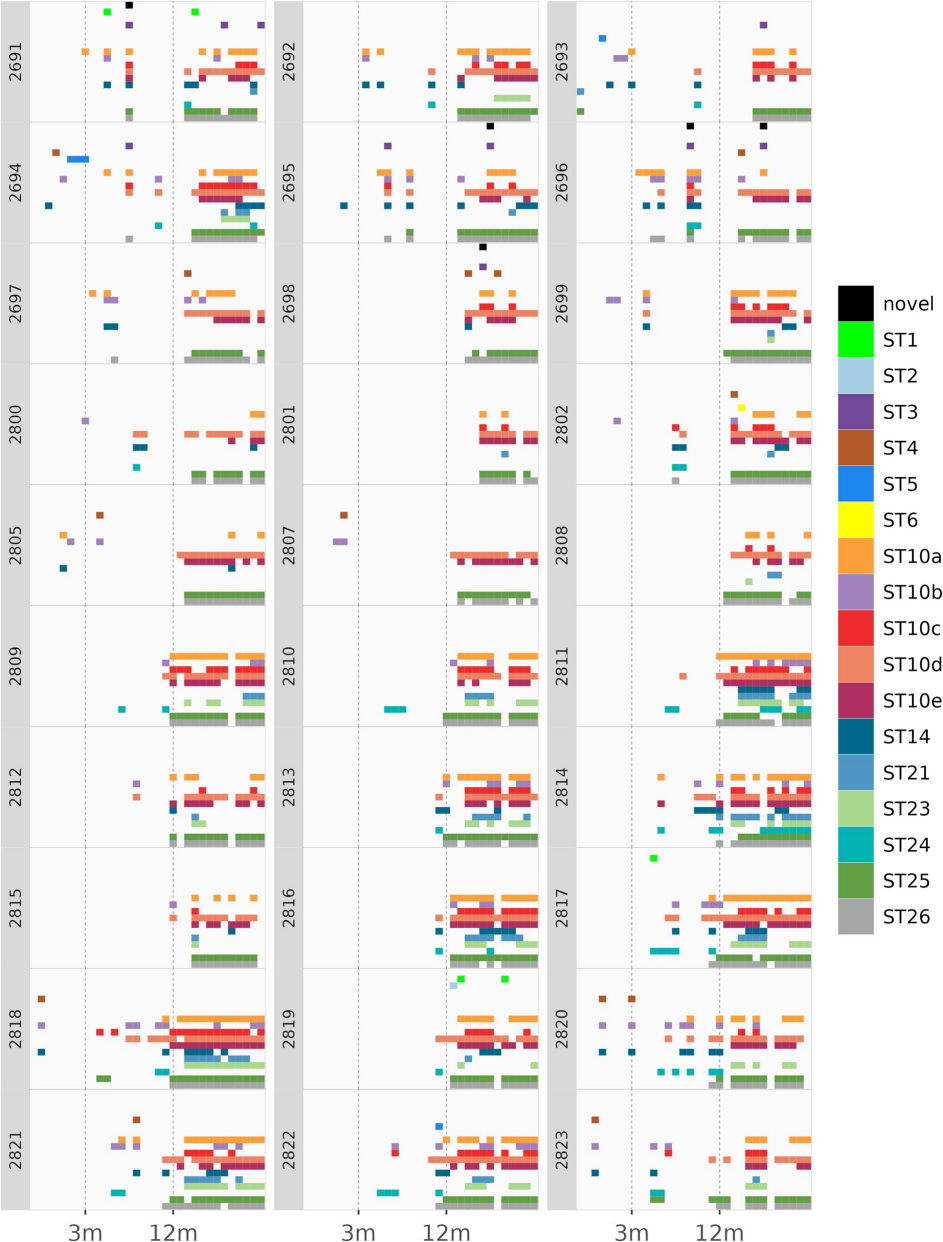
*Blastocystis* subtype diversity within each dairy heifer calf throughout the 24-month study period**.** Plot was generated using R packages dplyr v1.0.10 and ggplot2 v3.4.0

With the exception of ST10, no samples were found to contain multiple variants of the same ST. However, multiple sequence variants of ST10 were frequently observed in the same sample (Fig. 2). Additionally, a great deal of genetic diversity was observed among sequences classified as ST10 with as little as 91.8% and as much as 98.3% shared identity between ST10 sequence variants. To allow for a deeper investigation into the longitudinal relationships within ST10, subgrouping of ST10 sequence variants was performed based on sequence similarity and phylogenetic clustering among all 31 ST10 sequences (Additional file 1: Figure. S1). This enabled ST10 to be subdivided into five subgroups denoted as ST10a, ST10b, ST10c, ST10d, and ST10e. The ST10 subgroups displayed varying levels of diversity ranging from two to 17 unique genetic variants within each subgroup (Table 2). While individual samples were found to contain between one and five different subgroups, two variants of the same subgroup were not observed within the same sample, supporting the use of the five ST10 subgroups for exploring longitudinal relationships in this study.

Subgroups ST10d and ST10e had the most sequence differences compared to the other ST10 subgroups, with as little as 91.8% shared identity between ST10e and ST10a and as little as 92.3% shared identity between ST10d and ST10a. Given the large number of differences between ST10d and ST10e and other ST10 subgroups, it is likely that these sequences represent different subtypes.

### Mixed ST infections among dairy calves

Mixed infections with multiple STs present within the same sample were common among the study population (Fig. 2). Of the 437 positive samples in the study, 388 samples (88.8%) contained more than one ST. Among samples containing multiple STs, there was an average of 3.2 STs and 4.7 genetic variants per sample and a maximum of seven STs and 11 genetic variants observed within an individual sample. Of the 49 samples with only one ST, seven contained two genetic variants representing different variants of ST10. The post-weaned age group had the most single infections, with 25 positive samples within this age group containing only a single ST (Fig. 2). There were 18 and six single infections present in the pre-weaned and heifer groups, respectively. Over the 24-month study period, individual calves were observed to host a variety of STs (Fig. 2). The number of STs observed in an individual calf over the full study period ranged from four to 10, and the genetic variant count ranged from nine to 23.

### Longitudinal observations of *Blastocystis* STs among dairy calves

**Fig. 3:**
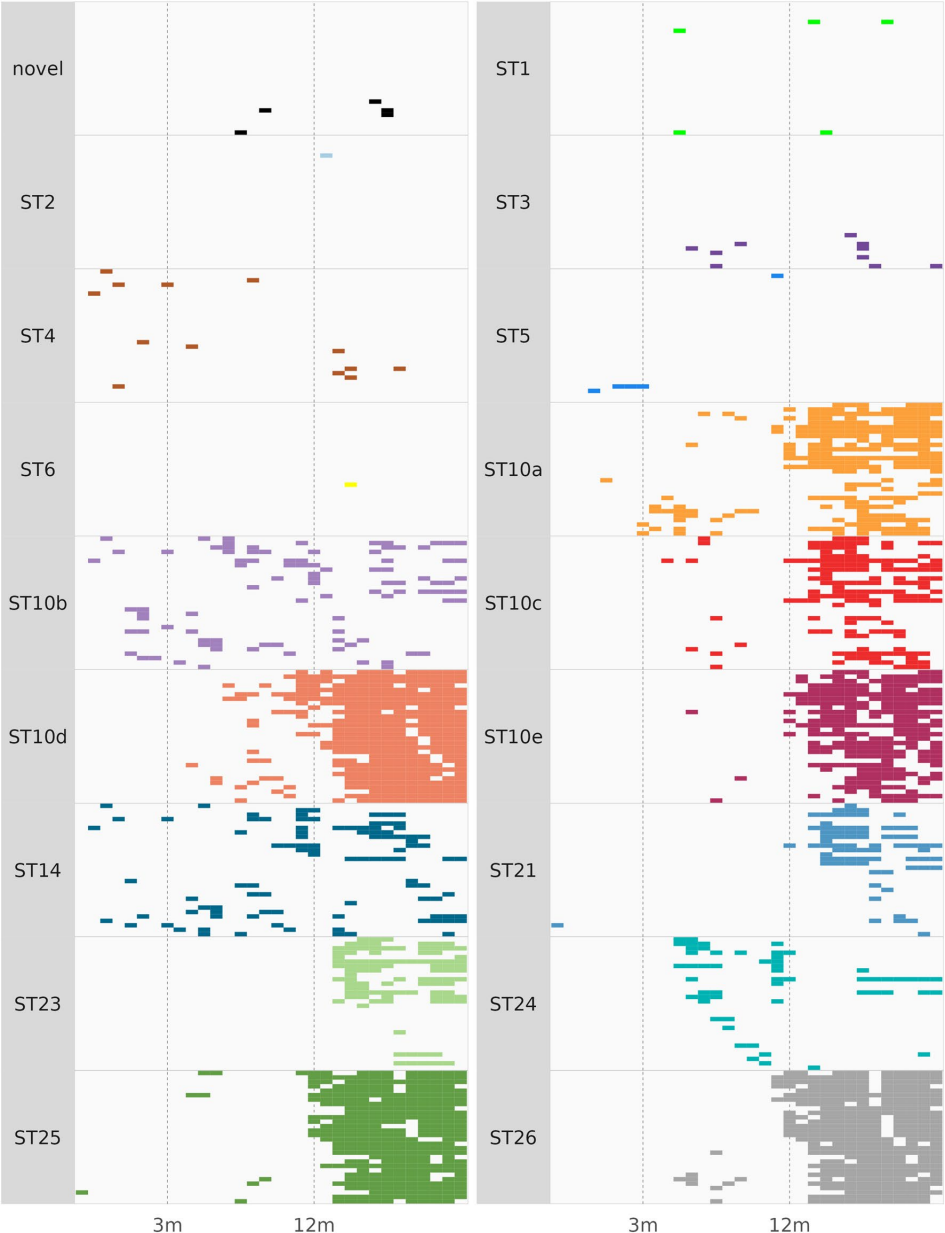
*Blastocystis* subtype distribution among dairy heifer calves by individual over the 24-month study period. Plot was generated using R packages dplyr v1.0.10 and ggplot2 v3.4.0

Of the STs observed in this study, ST1–ST6 have been frequently documented in human and animal samples and will be referred to as zoonotic STs. ST10, ST14, ST21, and ST23–ST26 have been largely reported in animals and will be referred to as enzootic STs. The overall prevalence of each ST among *Blastocystis*-positive samples varied widely (Table 2). However, zoonotic STs were less common in the study population than enzootic STs and were observed in 0.2–3% of *Blastocystis*-positive samples (Table 2). ST2 and ST6 were the least common subtypes observed in the study and were both found in only one sample each in heifers that were 13 and 15 months of age, respectively (Tables 2, 3; Fig. 3). ST4 was the most common zoonotic ST observed in this study and was found in 13 samples obtained from 11 different calves ranging in age from 2 weeks to 19 months (Tables 2, 3, Fig. 3). ST1, ST3, and ST5 represented 1.1%, 2.3%, and 1.1% of positive samples, respectively. Similarly, the potentially novel ST was not common in dairy calves and was observed in 1.1% of positive samples. Enzootic STs were the most common STs in the study population, and the prevalence of enzootic STs among the *Blastocystis*-positive samples ranged from 12.8% to 92.7% (Table 2). ST24 was the least common enzootic ST, representing 12.8% of positive samples. ST14, ST21, and ST23 represented 22.2%, 16.7%, and 22.4% of *Blastocystis*-positive samples, respectively. ST25 and ST26 were observed in 73% and 74.4% of *Blastocystis*-positive samples, respectively. ST10 was the most common ST in the study population and was observed in 92.7% of positive samples.

**Table 3:** *Blastocystis* subtype observations and prevalence by age group in a longitudinal study of dairy heifer calves

Age group	No. of tested samples	No. of positive samples	Subtypes observed^a^	No. of observations	Percentage of positive samples in age group	Percentage of all samples in age group
Pre-weaned (1–8 weeks)	240	26	ST4	6	23.1	2.5
			ST5	4	15.4	1.7
			ST10a	3	11.5	1.3
			ST10b	14	53.8	5.8
			ST14	9	34.6	3.6
			ST21	1	3.8	0.4
			ST25	1	3.8	0.4
Post-weaned (3–11 months)	360	76	ST1	2	2.6	0.6
			ST3	4	5.3	1.1
			ST4	2	2.6	0.6
			ST5	1	1.3	0.3
			ST10a	24	31.6	6.7
			ST10b	33	43.4	9.2
			ST10c	9	11.8	2.5
			ST10d	39	51.3	10.8
			ST10e	3	3.9	0.8
			ST14	34	44.7	9.4
			ST24	39	51.3	10.8
			ST25	8	10.5	2.2
			ST26	13	17.1	3.6
			Novel	2	2.6	0.6
Heifer (12–24 months)	390	335	ST1	3	0.9	0.8
			ST2	1	0.3	0.3
			ST3	6	1.8	1.6
			ST4	5	1.5	1.3
			ST6	1	0.3	0.3
			ST10a	203	60.6	52.1
			ST10b	47	14.0	12.1
			ST10c	134	40.0	34.4
			ST10d	312	93.1	80
			ST10e	226	67.5	57.9
			ST14	54	16.1	13.8
			ST21	72	21.5	18.5
			ST23	98	29.3	25.1
			ST24	17	5.1	4.9
			ST25	310	92.5	79.5
			ST26	312	93.1	80
			Novel	3	0.9	0.8

^a^ST10 was divided into five subgroups (ST10a, ST10b, ST10c, ST10d, and ST10e)

The total number of STs present within the study population also varied by age (Table 3). Subtype diversity increased with age, and six STs were observed among the pre-weaned calves, including ST4, ST5, ST10 (ST10a and ST10b), ST14, ST21, and ST25. Nine STs were observed among post-weaned calves, including ST1, ST3, ST4, ST5, ST10 (ST10a, ST10b, ST10c, ST10d, and ST10e), ST14, ST24, ST25, ST26, and the potentially novel ST. The heifer age group was the most diverse, with 12 STs observed including ST1, ST2, ST3, ST4, ST6, ST10 (ST10a, ST10b, ST10c, ST10d, and ST10e), ST14, ST21, ST23, ST24, ST25, ST26, and the potentially novel ST. The highest ST count at an individual collection point was nine and was observed among samples collected at 14, 15, 18, and 19 months. Aside from the 10-week collection point, for which no positive samples were observed, the lowest ST count observed was two at 1, 5, 7, 12, and 20 weeks.

The prevalence of individual STs varied by age in the study population (Table 3; Fig. 3). Although zoonotic STs were observed in all three age groups in the study, they were more common in pre-weaned calves than in post-weaned calves or heifers (Table 3; Fig. 3). Zoonotic STs represented 38.5% (10/26) of *Blastocystis*-positive samples in the pre-weaned age group but only 11.8% (9/76) and 4.8% (16/335) of *Blastocystis*-positive samples in the post-weaned and heifer age groups. Interestingly, despite having the highest prevalence of zoonotic STs, the pre-weaned age group was the least diverse in terms of zoonotic STs among the age groups. Only two STs, ST4 and ST5, were observed in the pre-weaned age group, while four STs, ST1, ST3, ST4, and ST5, were observed in the post-weaned age group (Table 3). Four STs, ST1, ST2, ST3, and ST4, were also observed in the heifer age group. Zoonotic STs did not appear to cause chronic infection in dairy heifer calves in this study (Figs. 2, 3). Only ST5 was observed in consecutive samples from the same calf, with three consecutive samples from an individual calf (#2694) found to be positive for *Blastocystis* from 6 to 8 weeks of age (Fig. 2). All other zoonotic ST infections were sporadic, and even when the same zoonotic ST was observed in the same calf, multiple months passed between observations (Fig. 2).

Distinct age-associated patterns within the study population were observed for ST10 subgroups (Fig. 3). Although ST10a was present in all three age groups, there was a clear increase in prevalence associated with age (Table 3). The percentage that ST10a represented out of the *Blastocystis*-positive samples within an age group was 11.5% for the pre-weaned calves, increasing to 31.6 and 60.6% for the post-weaned calves and heifers, respectively (Table 3). ST10b was the most prevalent ST among pre-weaned calves, representing 53.8% of positive samples, but decreased to 43.4% and then to 14% in the post-weaned and heifer groups, respectively. ST10c was the least common ST10 subgroup overall, not present in pre-weaned calves, and observed in only 21.5% of all positive samples in the study population. ST10c appears to increase with age and represented 11.8% of positive samples within the post-weaned age group and increased to 40% in the heifer group. All ST10 subgroups displayed sporadic infection patterns in both pre-weaned and post-weaned calves (Fig. 3). However, by 12 months of age, ST10a appeared to cause chronic carriage in the study population, while ST10b and ST10c infections continued to appear more sporadically (Fig. 3). Subgroups ST10d and ST10e were also not observed in the pre-weaned calves (Table 3). ST10d represented 51.3% of positive samples within the post-weaned age group, and with ST24 was the most common ST within this age group. ST10d increased to 93.1% of positive samples in the heifer group and with ST26 was the most common ST observed in the heifer group. ST10e represented 3.9% of positive samples within the post-weaned age group and was the least common ST10 subgroup in this age group. However, ST10e was quite common among heifers, representing 67.5% of positive samples in this age group. Both ST10d and ST10e appear to cause chronic infection in the study population (Fig. 3).

ST14 was observed in all three age groups in the study (Table 3; Fig. 3). ST14 was the second most common ST in the pre-weaned and post-weaned age groups, representing 34.6% and 44.7% of positive samples, respectively. However, ST14 prevalence decreased in the heifer group, representing only 16.1% of positive samples in this age group. Interestingly, ST14 displayed a sporadic infection pattern in all three age categories (Fig. 3). While individual calves did test positive for ST14 at consecutive time points, ST14 infections did not persist beyond a stretch of 5 months in any individual calf.

ST21 and ST23 displayed a clear association with the heifer age group. ST21 was observed in a single pre-weaned calf and was not observed in any post-weaned calves. However, ST21 was observed in 72 positive samples in the heifer group, representing 21.5% of positive samples in this age group. Interestingly, ST21 infections appeared to differ in chronicity by host, with some calves testing positive over multiple consecutive time points, and others testing positive at only a single time point (Fig. 3). ST23 was observed in 29.3% of positive samples in the heifer group, and infection appeared to be chronic in most of the calves in which it was observed. Although gaps in positivity were observed, many ST23-positive calves carried this ST over long periods of time within the heifer age group (Fig. 3).

ST24 displayed an association with the post-weaned calves and was the most common ST in this age group (Table 3). ST24 was not observed in the pre-weaned calves, represented 51.3% of positive samples within the post-weaned age group, and represented only 5.1% of positive samples in the heifer group. This ST appeared to cause sporadic infections in the post-weaned age group. However, of the six calves which were positive for ST24 in the heifer group, one calf displayed a more chronic infection pattern consistently testing positive for ST24 over a period of several months (Fig. 3).

ST25 and ST26 displayed similar infection patterns in the study population. ST25 was observed in only a single sample in the pre-weaned age group, and ST26 was not observed in this age group. ST25 represented 10.5% of positive samples in the post-weaned age group, and ST26 represented 17.1% of positive samples in this age group. However, both ST25 and ST26 were among the most common STs observed in the heifer age group. ST25 represented 92.5% of positive samples in the heifer age group, and ST26 represented 93.1% of positive samples in the heifer age group. Like ST10d, both ST25 and ST26 appear to cause chronic infection in heifers with most calves testing positive at consecutive time points. ST25 and ST26 infections are sporadic in other age groups with individual calves testing positive at a maximum of two consecutive time points before infections drop off for several months between the post-weaned and heifer age groups (Fig. 3).

The potentially novel ST was not common in the study population and appeared to cause only sporadic infections (Table 3; Fig. 3). The potentially novel ST was not observed in the pre-weaned age group and was present in only 2.6% and 0.9% of positive samples in the post-weaned and heifer age groups, respectively.

### Association between age and presence of *Blastocystis*

Logistic regression analysis was used to investigate the associations between age groups and presence of *Blastocystis* (Table 1). Because zoonotic STs, ST1–ST6, were observed both infrequently and sporadically, they were pooled into a single category for the analysis. Because of the lack of ST21- and ST23-positive samples in pre-weaned and post-weaned calves, these STs were excluded from analyses. Also, excluded from the logistic regression analyses was the potentially novel ST as it was present in so few samples. While the logistic regression analysis comparing the pre-weaned calves with the older groups (post-weaned and heifers) did find that the odds of zoonotic ST infection decreased with age, the associations were not statistically significant (Table 1). When associations between ST10 subgroups and age categories were compared via logistic regression analysis, only ST10a and ST10b had significant associations with age (Table 1). Risk of ST10a infection increased significantly with age in both post-weaned and heifer groups; however, ST10b was significantly associated only with the heifer group. Despite ST14 being among the most common STs in both pre-weaned and post-weaned calves and less abundant relative to other STs in the heifer group, ST14 was observed to have statistically significant associations with age. The odds of ST14 infection increased in both the post-weaned and heifer groups (Table 1). Additionally, ST25 was significantly associated only with the heifer group. No other significant outcomes were observed for the individual STs included in the analysis.

Because most STs (ST21, ST23, ST24, ST25, ST26, potentially novel ST) and subgroups ST10c, ST10d, and ST10e were not observed in all age groups, a separate logistic regression analysis was performed comparing associations between ages < 12 months and ≥ 12 months (Table 4). When infection risk was assessed using only two age categories, risk of infection increased significantly with age for all STs and subgroups except for ST24 and combined zoonotic STs (Table 4).

**Table 4:** Logistic regression analysis comparing *Blastocystis* infection status between age categories < 12 months and ≥ 12 months

	Log odds	*P*-value	95% CI
*Blastocystis*	3.39	7.11E−78^c^	3.0, 3.7
Zoonotic subtypes^a^	0.27	0.437132	−0.4, 0.9
ST10a^b^	3.14	1.50E−45^c^	2.7, 3.6
ST10b^b^	0.48	0.028044^c^	0.1, 0.9
ST10c^b^	3.54	9.99E−24^c^	2.8, 4.2
ST10d^b^	4.05	3.44E−84^c^	3.6, 4.5
ST10e^b^	5.61	1.29E−21^c^	4.5, 6.8
ST14	0.73	0.000677^c^	0.3, 1.2
ST24	−0.42	0.156789	−1.0, 0.2
ST25	5.54	7.49E−54^c^	4.8, 6.2
ST26	5.20	5.27E−64^c^	4.6, 5.8

CI confidence intervals

^a^Zoonotic subtypes included ST1–ST6

^b^ST10 was divided into five subgroups (ST10a, ST10b, ST10c, ST10d, and ST10e)

^c^Statistically significant

## Discussion

Longitudinal data are necessary for understanding the biology and epidemiology of *Blastocystis*, but longitudinal studies following the same population from birth to adulthood have never been conducted for *Blastocystis*. To fill this data gap, we assessed infection prevalence and subtype diversity in a single population of 30 dairy calves from birth through 24 months of age. Prevalence and subtype diversity were measured via PCR and NGS, which allowed for assessment of both infection status and within-host subtype diversity, as this strategy has been shown to be suitable for assessing mixed-subtype infections within a single host [28]. Such a strategy is especially important in thoroughly assessing *Blastocystis* subtype diversity as mixed-subtype infections have been shown to be common in both humans and cattle, and their presence can hinder subtype identification [12, 29–31, 47]. Using an NGS strategy for exploring subtype diversity, we screened 990 fecal samples representing 33 individual samples per calf taken over the 24-month study period and have produced a comprehensive longitudinal study for *Blastocystis*.

In the study population, an overall prevalence of 44.1% was observed, and over the course of the study, all 30 calves tested positive for *Blastocystis*. The overall prevalence observed in cattle in this study is within the range reported in cattle in previous studies [30, 48, 49]. However, as no longitudinal data from cattle exist, this is the first study to demonstrate a cumulative prevalence of 100% for dairy cattle. The high overall prevalence and cumulative prevalence of *Blastocystis* in this study further confirm that *Blastocystis* is a common parasite of dairy cattle.

When considered by age group (pre-weaned, post-weaned, or heifer), *Blastocystis* prevalence was observed to increase significantly with age from 10.8% in pre-weaned calves to 85.9% in heifers (Fig. 1; Table 3). Associations between *Blastocystis* prevalence and age have been observed in other human and animal studies, although the factors driving this relationship are not known [29, 41, 42, 50]. One hypothesis is that young mammals are less likely to be exposed to *Blastocystis* while milk consumption is their main form of nutrition. Although exposure is likely a factor influencing the prevalence of *Blastocystis* in dairy calves, the results of this study suggest that physiology may be an important factor as well. Prevalence was lowest in the pre-weaned age group in this study, yet the post-weaned age group also had a relatively low overall prevalence of 21.1% despite living in close quarters with other cattle, which could facilitate high rates of transmission and reinfection. Indeed, both pre-weaned and post-weaned calves display sporadic infection, which could indicate that infection in these age groups is not chronic and likely relies upon re-exposure to facilitate infection (Fig. 2). Heifers display both high prevalence and chronic infection patterns, which may support the hypothesis that biological differences are key in determining infection chronicity in cattle.

Point prevalence data from this study indicate that while infection increases with age, infection status is dynamic in cattle over time. Large decreases in prevalence were observed at two time points, when calves were 3 months and 8 months of age, where prevalence dropped to 0% and 10%, respectively (Fig. 1). The absence of any positive samples from the 3-month age category is particularly intriguing as calves in the study population are undergoing major transitions in diet and lifestyle at this age. By 3 months, calves are no longer being bottle-fed milk and are transitioned to group housing. The absence of *Blastocystis* in dairy calves at this time point could indicate that factors such as changing gastrointestinal physiology, diet, or stress can significantly impact infection status. Little data on the influence of such factors on infection status exist. However, it has been reported that diet can significantly influence *Blastocystis* prevalence in cattle where prevalence increased in cattle consuming corn silage but decreased in cattle consuming pasture grass [48]. These findings do not align with the observations of the present study in which calves in pens had a lower prevalence than those on pasture, which may indicate that age-related physiological differences have a stronger influence than diet on *Blastocystis* infection. The fact that the transition to group housing does not yield an immediate and large increase in prevalence further supports the idea that physiology may be an important factor driving infection prevalence. It is not until after 9 months that infection prevalence steadily increases in the study population (Fig. 1). In fact, from 9 to 15 months, there is consistent growth in infection prevalence. Following this period of growth, prevalence rates of 90–100% are maintained in the study population through the end of the study at 24 months of age. The high prevalence and constant presence of *Blastocystis* in the adult cattle in this study could indicate that it is not until adulthood that cattle are suitable hosts for long-term colonization with *Blastocystis*.

Data from age-related prevalence associations with *Blastocystis* infection in humans report similar trends, with prevalence in children being lower than in adult age groups [29, 41, 42]. Because no longitudinal studies in humans have followed the same population over the same developmental time scale, it is hard to know whether physiological milestones in humans are associated with shifts in infection prevalence. Humans and cattle both exhibit periods of flux in microbiome composition and diversity linked to age-related shifts in both diet and physiology [51, 52]. Thus, it is likely that the infection dynamics of *Blastocystis* are related to these factors for both humans and cattle. Defining such relationships is a complicated task, and future studies which aim to unravel the influence of host diet and physiology on *Blastocystis* infection or colonization are needed to answer these difficult but fundamental questions.

There was a great deal of subtype diversity in the study population with 13 named STs and one potentially novel ST observed throughout the study period (Table 2). Such diversity may be a hallmark of ruminants as other NGS-based studies of *Blastocystis* ST diversity in cattle and other ruminants have also identified high levels of ST diversity. In a recent study of dairy calves from the United States, which also employed NGS for ST differentiation, 14 STs were observed among 75 *Blastocystis*-positive calves [28]. Similarly, 10 STs were reported in 108 *Blastocystis*-positive cattle from northern Spain using NGS [30]. A study of domestic animals from Colombia used NGS to identify nine, eight, and six STs in eight cattle, two goats, and one sheep, respectively [12]. In wild white-tailed deer from the United States, a wide diversity of STs were identified using NGS, with 12 STs identified in 71 *Blastocystis*-positive deer [11]. On a global scale, previous studies of ST diversity in cattle have documented 17 different STs from cattle populations [1, 12]. However, the diversity of subtypes observed in the present study is especially intriguing. While the aforementioned studies included animals and samples from multiple populations of the same host, the study of dairy calves presented here constitutes a single population living in the same location under controlled conditions. Yet even under these circumstances 14 different STs were observed, providing evidence that a single population can be host to a wide variety of *Blastocystis* STs over time.

By measuring ST diversity in dairy calves from birth through 24 months of age, this study was able to explore the associations of *Blastocystis* STs within three age groups: pre-weaned calves, post-weaned calves, and heifers. Subtypes which are considered zoonotic (ST1–ST6) because they are commonly reported in humans and animals were observed in all three age groups in the study population (Table 3). However, zoonotic STs were most common among pre-weaned calves and represented 38.5% of positive samples in the pre-weaned age group. Among post-weaned and heifer age groups zoonotic STs represented 11.8% (9/76) and 4.8% (16/335) of positive samples, respectively. Zoonotic STs were also found to be among the most common STs in pre-weaned dairy calves from across the United States [28, 50]. Zoonotic STs are reported in other large-scale studies of adult cattle, but as in the present study, zoonotic STs are less common among older cattle [30, 48, 49]. Such observations may indicate that pre-weaned cattle could more commonly serve as reservoirs for zoonotic ST transmission. Future studies of zoonotic STs in this age group of cattle as well as other pre-weaned ruminants could help to clarify their role in the transmission of *Blastocystis*.

Age-related associations were also observed for ST14, which was one of the most common STs in the pre-weaned and post-weaned groups. The differences in ST14 by age group are particularly interesting, as it has been suggested that ST14 is a cattle-adapted ST [49]. However, ST14 represented only 22.2% of all *Blastocystis*-positive samples in this study and displayed a sporadic infection pattern throughout the study period (Table 1; Fig. 3). Thus, the present study may indicate that while cattle are commonly exposed to this ST, they may not represent the primary host for ST14. Additional factors such as age, diet, or environmental exposure may need to be considered in understanding the true relationship between cattle and ST14.

ST24 displayed a strong age-associated relationship with the post-weaned group and was the most common ST in this age group along with ST10d (Table 3). Like ST10b and ST14, the shifts in ST24 prevalence may be related to environmental exposure or other factors associated with lifestyle in the study population. As with ST14, ST24 has been reported in cattle in varying prevalence and was observed in 25.9% of adult cattle from Spain but in only 2.4% of pre-weaned dairy calves from the United States [30, 50]. Interestingly, 77.5% of positive samples from white-tailed deer in Maryland, the same state in which the present study was conducted, were found to contain ST24 [11]. Thus, exposure to wild ruminants may be a factor influencing ST diversity in domestic cattle. However, more comprehensive studies designed to investigate the relationship between domestic animals and co-inhabiting wildlife are needed to understand such relationships for cattle and other animal hosts.

Subtypes ST21, ST23, ST25, and ST26 had strong age-associated relationships with the heifer group. Like ST24, they were common in adult cattle from Spain and present but uncommon among pre-weaned calves from the United States [30, 50]. As in the present study, ST25 and ST26 were among the most common STs observed in adult cattle in Spain [30]. Interestingly, ST21 and ST23 were observed in similar prevalence rates among white-tailed deer from Maryland, but ST25 and ST26 were among the least common STs in white-tailed deer [11]. Thus, while ST21 and ST23 are common in cattle, their presence may be more likely due to environmental exposure, whereas ST25 and ST26 may be well adapted to persistent colonization of cattle. However, more data on such potential associations are needed to conclusively demonstrate such a relationship.

ST10 was the most common ST observed among calves in this study. This finding is consistent with studies worldwide where ST10 is the most common ST reported in cattle [1]. ST10 also had the most intra-subtype diversity similar to what was observed in pre-weaned dairy calves from the United States, where ST10 also had the most unique genetic variants in the study [50].

Using ST10 subgroups, age-related patterns were observed among calves (Tables 3, 4; Fig. 3). Differences in prevalence and infection chronicity between ST10a and ST10b are particularly intriguing as these two subgroups share > 97% sequence similarity across the sequenced gene region. Yet, despite this genetic similarity, the life history of these two subgroups within dairy calves over time is quite different. In fact, ST10b is unique among the ST10 subgroups in being the only subgroup which is not highly represented in the heifer group. Thus, ST10b may have genetic differences which predispose it to a different reservoir of infection or give it a disadvantage for surviving in the gut of cattle as they age. Exploring the relationship between ST10 subgroups and other factors such as diet, physiology, and other potential reservoir hosts may assist in understanding the potential importance and drivers of infection differences.

Dividing ST10 into multiple subgroups provided important insights into the longitudinal relationships between cattle and ST10. However, whether such divisions should be translated into new subtype designations will require more data including full-length reference sequences for each subgroup. Based on the gene region used in this study, sequence identity between ST10 subgroups varies from 91.8% shared identity between ST10a and ST10e to up to 98.3% between ST10d and ST10e. While we withhold assigning new ST designations to any ST10 subgroup in this study, the data presented here indicate that ST10d and ST10e likely represent a different subtype. Although more data are needed to fully explore the importance of the genetic diversity within ST10, data from this study indicate that this diversity may be associated with host specificity or infection potential in cattle of different ages. Future studies which seek to explore this diversity and produce the data needed for a revision of this subtype will be crucial to answering such questions. Given that ST10 has now been identified in humans, the importance of describing and understanding the genetic variability and epidemiology of this subtype is of immediate relevance [20, 23].

There was a single potentially novel ST observed among cattle in this study. This ST was observed among five samples and was represented by two unique genetic variants (Table 2). The nucleotide sequences of the potentially novel ST differed by ≥ 3% from any known ST. However, without the full-length sequence of the *SSU *rRNA gene for the potentially novel ST, promotion to subtype status within the subtype numbering system currently in use in the field of *Blastocystis* is being withheld [15, 53]. Comparisons between the approximately 480-bp sequence generated in this study and sequences available in GenBank did reveal a sequence in GenBank which shares 98.7–99.1% sequence identity with the genetic variants of the potentially novel sequence. Interestingly, this sequence (GenBank Accession no. MW301904) came from a child, and while it was reported as ST3, it shares only 96% sequence identity by BLAST with the closest match in GenBank [54]. Given that the potentially novel ST is most like a sequence from a human and that it was observed only sporadically in cattle, it is possible that the potentially novel ST is of anthropogenic origin and could be a zoonotic infection risk. If full-length reference sequences can be obtained from future identification of samples containing this potentially novel ST, it will help to clarify the status of this potential subtype and study its epidemiology.

In this study, cattle were hosts for a remarkably diverse group of *Blastocystis* STs. Within-host diversity increased with age, and individual calves served as hosts for up to 10 STs over the course of the 24-month study period (Fig. 2). No study has sampled the same host over as many time points or over as long a physiological time span as was achieved with this study. However, comparisons can be made to the few longitudinal studies from humans which do exist. For example, humans and cattle seem to share a common pattern of increasing prevalence with age [33]. There are also data suggesting that for some adult humans, *Blastocystis* colonization is temporally stable, while for others it is not [32, 34, 55]. Additionally, a recent report indicates that temporal stability of *Blastocystis* may occur in children as well. In a year-long survey of toddlers from Spain where samples were collected bimonthly, five children were observed to carry the same genetic variant of *Blastocystis* at multiple time points, and three children were found to be positive for the same genetic variant at every bimonthly sample collected in the study period [35]. In the present study, infection chronicity in cattle varied widely by ST, and even within ST differences in chronicity were observed. Relationships between infection chronicity and ST diversity for humans remain to be explored. However, data from this longitudinal study of cattle support the idea that genetic diversity, even beyond the ST level, is an important factor to consider in such studies.

## Conclusions

Cattle, like humans, are host to a wide variety of *Blastocystis* STs. Obviously, longitudinal studies in cattle cannot be directly translated to expectations of longitudinal trends in humans. However, the data from this study can be used to inform our understanding of how differences in prevalence and ST diversity may be driven by age, host physiology, and environment. This study did not investigate the relationship between the presence of other eukaryotic or prokaryotic members of the gut and *Blastocystis*. However, it is certainly feasible that the microbial diversity of the gut including the presence of different *Blastocystis* STs could influence susceptibility to infection or colonization with any individual ST. Pairing NGS strategies which are capable of exploring the diversity within the gut of a host over time with longitudinal studies of *Blastocystis* in different hosts from a variety of environmental settings will help to answer questions about internal and external factors influencing *Blastocystis* infection. Such studies, although difficult to conduct, will be essential to better understand the complex epidemiology of *Blastocystis* and conditions under which it may serve as a commensal, a parasite, or a pathogen.

## Supplementary Information

**Additional file 1: Fig. S1.** Phylogenetic tree of all 31 unique sequence variants of ST10. Accession number of each ST10 sequence variant are used in the tree, and ST10 subgroups (ST10a–ST10e) are identified.

## Data Availability

Sequence data generated in this study were submitted to the GenBank database under the accession numbers OQ298847-OQ298915. Raw FASTQ files were submitted to NCBI’s sequence read archive under project PRJNA927016 and accession numbers SRR23210482-SRR23210918.

## Abbreviations

ST: Subtype
*SSU* rRNA: Small subunit ribosomal RNA
PCR: Polymerase chain reaction
NGS: Next-generation amplicon sequencing
