# Supplementary figures and images for: A longitudinal study of Blastocystis in dairy calves from birth through 24 months demonstrates dynamic shifts in infection rates and subtype prevalence and diversity by age
Source: Parasit Vectors. 2023 Jun 2;16:177. doi: 10.1186/s13071-023-05795-0 (PMC10236725; doi:10.1186/s13071-023-05795-0)

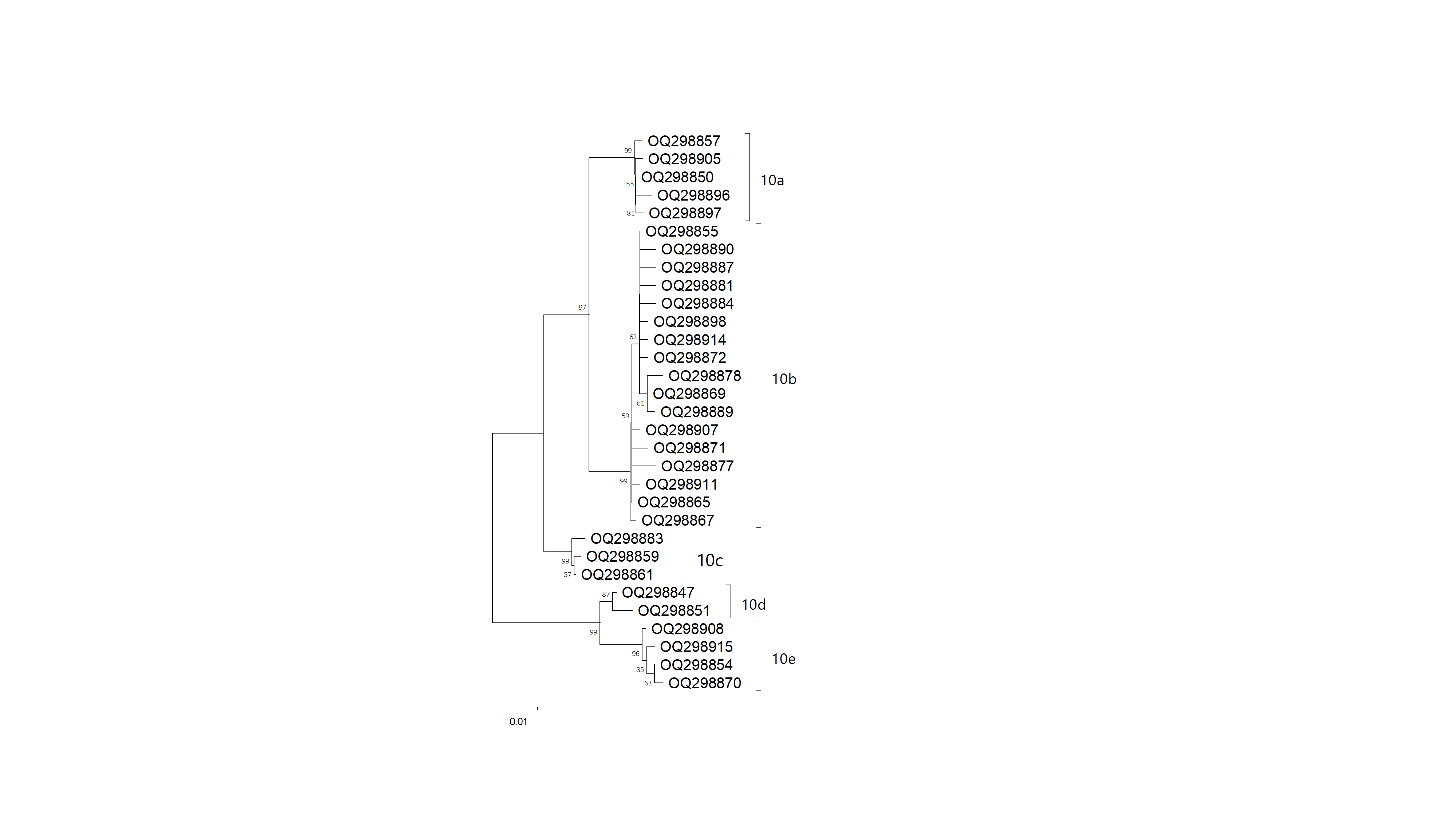

Supplement: Supplementary file 1 — Additional file 1: Fig. S1. Phylogenetic tree of all 31 unique sequence variants of ST10. Accession number of each ST10 sequence variant are used in the tree, and ST10 subgroups (ST10a–ST10e) are identified. [file 13071_2023_5795_MOESM1_ESM.tif]
